# Fatal pulmonary thromboembolism after Achilles tendon open repair: A rare case report

**DOI:** 10.1097/MD.0000000000008887

**Published:** 2017-11-27

**Authors:** Dong Il Chun, Sanghyeon Lee, Sung Hun Won, Jaeho Cho

**Affiliations:** a Department of Orthopedic Surgery, Seoul Hospital, Soonchunhyang University College of Medicine, Seoul; b Department of Orthopaedic Surgery, Chuncheon Sacred Heart Hospital, Hallym University, Chuncheon, Republic of Korea.

**Keywords:** Achilles tendon rupture, pulmonary thromboembolism, venous thromboembolism

## Abstract

**Rationale:**

The operative procedure for Achilles tendon rupture is relatively simple, but venous thromboembolism is serious complication with a high incidence after Achilles tendon rupture. However, the guideline for thromboprophylaxis in Achilles tendon rupture is unclear.

**Patient concerns:**

The patient was 32-year-old male and underwent Achilles tendon open repair surgery. He was healthy and there are no abnormal findings other than Achilles tendon rupture. At 3 weeks after operation, the episode of loss of consciousness with convulsive movement occurred. The next day, suddenly cardiac arrest occurred.

**Diagnoses:**

Extensive pulmonary thromboembolism in both pulmonary arteries was identified in chest computed tomography and thrombus was also identified at the left popliteal vein on ultrasonography.

**Intervention:**

Anticoagulant therapy with heparin sodium was performed to manage the pulmonary thromboembolism.

**Outcomes:**

Brain swelling after ischemic brain damage, acute kidney injury, and pneumonia gradually occurred and aggravated. His condition became worse and he died about 2 weeks after the cardiac arrest episode.

**Lessons:**

Although the incidence of venous thromboembolism in Achilles tendon rupture is higher than that in lower leg injury patients, guideline for thromboprophylaxis is unclear. We suggest that thromboprophylaxis for Achilles tendon rupture should be considered and appropriate guidelines should be established.

## 1 Introduction

Achilles tendon rupture occurs at the age of 30 to 39 years with the highest incidence and the incidence is gradually increasing.^[1]^ Recently, both operative and nonoperative treatments are possible for Achilles tendon rupture. Operative treatment includes open or percutaneous repair and nonoperative treatment includes cast-immobilization or keeping functional brace. Operative treatment is a relatively simple procedure, but complications such as surgical wound infection, delayed wound healing, and adhesion may occur, while re-rupture may occur as a complication in nonoperative treatment.^[2]^ Among the complications, venous thromboembolism (VTE), including deep vein thrombosis (DVT) and pulmonary thromboembolism (PTE) is serious one with increased risk due to immobilization and non-weight bearing of injured lower limb after Achilles tendon rupture.^[3]^ Although incidence of DVT with regard to Achilles tendon rupture is greater than cases of lower leg injury or surgery^[4]^ and PTE was rarely reported,^[5–7]^ guidelines for thromboprophylaxis have not recommended and its evidence is unclear.^[8]^ This case demonstrates the rare fatal PTE case in a patient with no risk factor of VTE who underwent open repair after Achilles tendon rupture.

## 2 Case presentation

**Figure 1:**
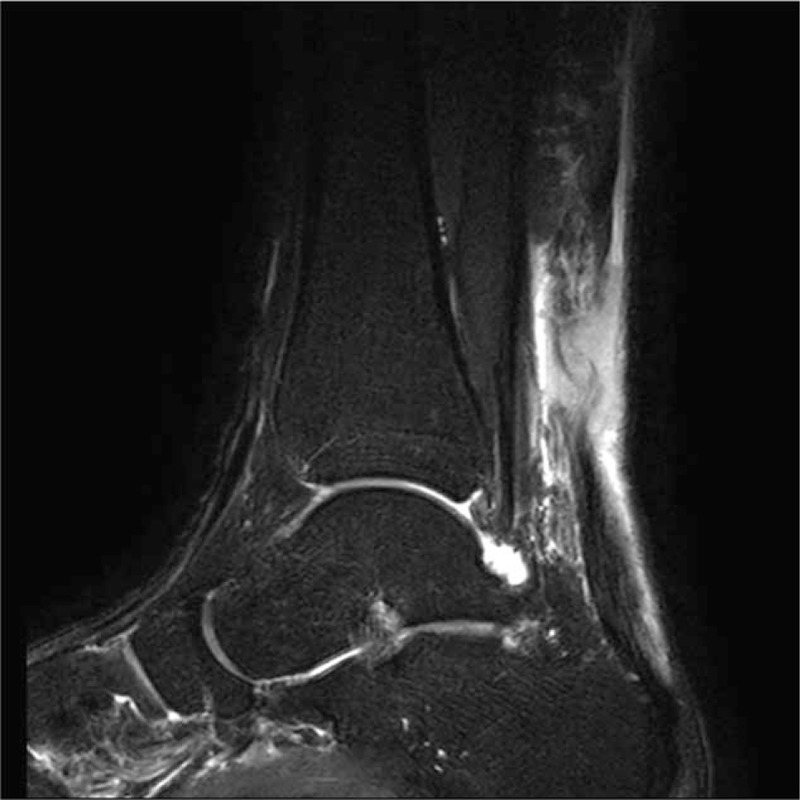
An ankle MRI image showing rupture of the Achilles tendon. On the fat-suppressed T2-weighted sagittal image, there is disruption of the Achilles tendon with a gap of about 4 cm at the upper 6 cm of the Achilles tendon calcaneal insertion.

A 32-year-old man visited the emergency room complaining left ankle pain, which developed suddenly with a pop sound behind the ankle during playing soccer. The pain was on posterior side of left ankle and also there was tenderness at the same site. The result of Thompson squeezing test was positive and Dimpling sign was identified at 6 cm above the Achilles tendon calcaneal insertion. There were no other symptoms and no abnormalities were found in the blood laboratory test. He was 170 cm tall, weighing 83 kg, and the body mass index (BMI) was 28.72 kg/m^2^. Also, he had no underlying disease, was a social drinker, did not smoke, and regularly exercised. No cardiovascular disease history existed with his family and relatives. An ankle magnetic resonance imaging (MRI) identified rupture of the Achilles tendon with a gap of about 4 cm at the upper 6 cm of the Achilles tendon calcaneal insertion, and confirmed that there was no specific finding including the vascular condition other than swelling around the ankle joint (Fig. 1).

Next day, the patient underwent an Achilles tendon repair operation under general anesthesia with prone position. Tourniquet was inflated at his proximal thigh with 350 mm Hg pressure for 35 minutes. A longitudinal incision was made just medial to the tendon. After the careful dissection, ruptured Achilles tendon was exposed. The Achilles tendon was restored by the Krachow method with absorbable surgical suture materials (Vicryl and Polysorb; ETHICON, Johnson & Johnson, Seoul, Korea) and the paratendon was repaired. After the irrigation with a normal saline, a layer by layer closure was performed and a short leg splint was applied with ankle plantar flexion position. After the operation, the patient recovered from anesthesia without any problems and allowed wheelchair and clutch ambulation. On the second postoperative day, the wound was clean and the patient did not complain of other symptoms other than the pain of wound, and on the sixth postoperative day, patient was discharged with short leg splint and he ambulated using a clutch. On the 14th postoperative day, he visited the outpatient clinic. The wound was healed completely, so we removed stitches. He was also allowed to perform full weight-bearing as tolerated, using a functional brace.

**Figure 2:**
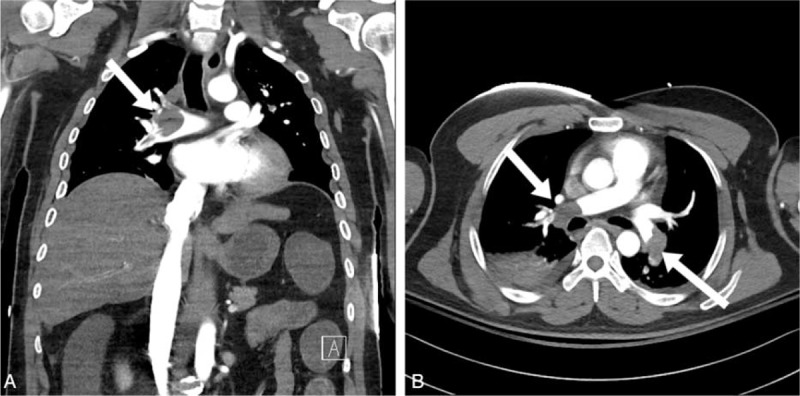
Chest computed tomography scan showing extensive bilateral pulmonary embolism in both pulmonary arteries. (A) Coronal image, (B) axial image.

**Figure 3:**
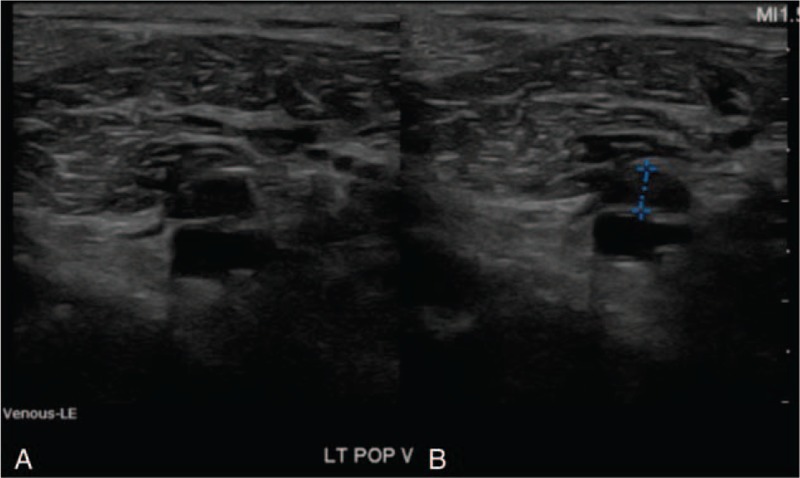
Color Doppler ultrasonography images of patient's left popliteal vein. (A) Popliteal vein and artery without compression; (B) Popliteal vein and artery with compression. Left popliteal vein is not compressible due to thrombus.

At 3 weeks after operation, he collapsed suddenly with loss of consciousness; after a few seconds later, he was standing up from the chair, and then sudden onset of convulsive movement was followed. It was his first attack. He was admitted to the department of neurology to work up about the episode of loss of consciousness. No specific findings were observed in the blood laboratory test result at the time of admission, so we planned to do neurologic evaluation including electroencephalogram (EEG) and brain MRI. There was no problem at the operation site and his lower leg. The episode of loss of consciousness with convulsive movement occurred again at the next day, and right after the episode, suddenly cardiac arrest occurred and cardiopulmonary resuscitation (CPR) was initiated. Return of spontaneous circulation was confirmed after 40 minutes of CPR. The work up to find the cause of deterioration of his condition was initiated, and hypoxic brain damage was identified in brain computed tomography (CT). Also, extensive PTE in both pulmonary arteries was identified in chest CT (Fig. 2) and thrombus was identified at the left popliteal vein on ultrasonography (Fig. 3). Two rounds of the episode of loss of consciousness were presumed to be due to hypoxic ischemic encephalopathy, and recovery of first episode was presumed to be due to recanalization of PTE. Anticoagulant therapy with heparin sodium was performed to manage the PTE, but brain swelling after ischemic brain damage, anuria, and pneumonia gradually occurred and aggravated. His condition became worse and he died about 2 weeks after the cardiac arrest episode.

## 3 Discussion

The VTE is a fatal complication in patients undergoing orthopedic surgery, especially in patients with nonweight-bearing and immobilization periods after a lower extremity injury or operation. In addition, the incidence of VTE in patients with Achilles tendon rupture is not low. In prospective study of 100 patients with acute Achilles tendon rupture over a 3-year period, DVT occurred within 2 months after Achilles tendon rupture in 34% patients and there were no significant differences in incidence between operative treatment and nonoperative treatment group.^[9]^ Also, in other study of incidence of symptomatic DVT after Achilles tendon rupture, a symptomatic DVT was developed in 27 of 115 patients (23.4%) after Achilles tendon rupture.^[3]^

According to the systemic review article,^[10]^ there are risk factors for VTE in patients undergoing foot and ankle surgery, including age over 60 years, obesity (BMI more than 30 kg/m^2^), immobilization, medical comorbidities, prior history of DVT, and oral contraceptive pills. In our case, we presented Achilles tendon rupture patients followed asymptomatic DVT and fatal PTE. Although the patient did not have any risk factor for VTE and he started ambulation after 2 weeks postoperatively, asymptomatic DVT and fatal PTE occurred. In the literatures’ review, there was only 1 case report about fatal PTE in patients with Achilles tendon rupture. However, in that case, PTE was diagnosed by autopsy after the patients died, and the patient underwent delayed operation that was performed 5 weeks after injury, so the authors suggested the possibility that asymptomatic DVT may have occurred before surgery.^[6]^ Therefore, we first report a rare case of fatal PTE after acute Achilles tendon rupture in healthy young patients with no risk factor for VTE.

According to meta-analysis investigating incidence of VTE in the foot and ankle surgery, the incidence of VTE in isolated foot and ankle surgery was 0.6% in the clinically assessed group, and 12.5% in the radiological evidence assessed group. On the contrary, the incidence of VTE in Achilles tendon rupture was 7% in the clinically assessed group, and 35.3% in the radiological evidence assessed group.^[4]^ High incidence of VTE in Achilles tendon rupture patients may be caused that loss of function of posterior compartment muscle of the lower leg, because gastrocnemius and soleus muscle were directly involved in injury of Achilles tendon. The foot, calf, and thigh muscle pump actions occupy most of the venous return at the lower extremities, among which the calf muscle pump occupies the largest capacity with an ejection fraction of 65%. The action of this pump with valves occurs when the deep fascia of the leg constrain the muscle at contraction; this process generates the high pressure of the muscular compartment.^[11]^ In patients with Achilles tendon rupture, the loss the function of calf muscle pump occurs in the both postoperative and nonoperative treatment, because splint or brace with ankle plantarflexion position is generally applied. So, the pressure of the posterior muscular compartments does not rise and it makes the venous return volume of the lower extremity decrease and the blood flow velocity decrease. Thus, the tendency for thrombus formation to occur in the vein valve pocket is increased.^[12]^ Therefore, compared with other lower leg injuries, the loss of function of calf muscle pump may be considered to be greater in Achilles tendon rupture patients. Considering the above-mentioned studies, we suggest that VTE after Achilles tendon rupture should be considered more deeply than lower leg injury and guideline for thromboprophylaxis after Achilles tendon rupture should be established.

There have been many studies on VTE that occurred after performing major operation of orthopedic surgery (knee and hip arthroplasty, hip fracture surgery). The American College of Chest Physicians (CHEST) recommended guidelines for prevention and management of VTE after performing major operation of orthopedic surgery and the evidence of these guidelines are relatively clear.^[8,13–16]^ However, they suggested no thromboprophylaxis for prevention of VTE after performing isolated lower leg injuries.^[8]^ In the thromboprophylaxis-related study of the Achilles tendon rupture, chemo-thromboprophylaxis did not show any significant difference between the applied and nonapplied groups. In a randomized placebo-controlled study that evaluated the effect of thromboprophylaxis on dalteparin in patients with Achilles tendon rupture, there was no difference in DVT incidence between thromboprophylaxis group (dalteparin) and placebo group.^[17]^ Also, Calder et al^[4]^ suggested that chemical prophylaxis using low molecular weight heparin (LMWH) was no benefit after foot and ankle surgery. However, the authors noted that specific measure such as IPCD (intermittent pneumatic compression device) could be helpful for thromboprophylaxis in the Achilles tendon rupture. In a recent study, DVT incidence was 21% in the IPCD-treated group and 37% in the control group, thus confirming the significant prevention of DVT after Achilles tendon rupture.^[18]^ Moreover, chemical thromboprophylaxis such as LMWH after orthopedic surgery may cause wound bleeding, cardiovascular adverse events, and wound-related infections.^[19]^ In addition, it is thought that the effect of chemo-thromboprophylaxis after Achilles tendon rupture can be further reduced due to stasis of blood flow caused by loss of function of the calf muscle pump. Therefore, we suggest that applying mechanical thromboprophylaxis such as IPCD may help to prevent DVT in Achilles tendon rupture.

## 4 Conclusion

In this case report, we first present a rare case of fatal PTE after acute Achilles tendon rupture in healthy young patients with no risk factor for VTE. Although the incidence of VTE in Achilles tendon rupture patients is higher than that in lower leg injury patients and may be fatal enough to cause patient death, the guideline for thromboprophylaxis in Achilles tendon rupture patients is unclear. We suggest that thromboprophylaxis for Achilles tendon rupture should be considered and appropriate guidelines should be established. In addition, applying mechanical thromboprophylaxis may be helpful in the thromboprophylaxis for Achilles tendon rupture.
